# Economic burden of MDR/RR-TB and implementation of a treatment subsidy policy in Lishui City, China: a retrospective study following strobe guidelines

**DOI:** 10.1186/s12913-026-14998-x

**Published:** 2026-06-23

**Authors:** Qian Zhu, Guang-Nao Zhou, Jing Guo

**Affiliations:** Department of Tuberculosis, Lishui Hospital of Traditional Chinese Medicine, No.800 of Zhong Shan Street, Liandu District, Lishui, 323000 China

**Keywords:** Anti-TB drugs, Burden, Direct medical expenses, Multidrug-resistant tuberculosis, Rifampicin-resistant tuberculosis

## Abstract

**Background:**

Multidrug-resistant and rifampicin-resistant tuberculosis (MDR/RR-TB) imposes significant financial burdens on patients. This study aimed to examine the current status of the economic burden of disease in patients with MDR/RR-TB in Lishui City, Zhejiang Province, and to describe the actual implementation status of the diagnosis-and-treatment subsidy policy and explore associations between subsidy indicators and patient treatment outcomes.

**Methods:**

We conducted a retrospective survey of 117 MDR/RR-TB patients diagnosed and managed in Lishui City from 2017 to 2022. Data on sociodemographic characteristics, diagnosis and treatment processes, medical expenses, and subsidy information were collected and analyzed using questionnaires.

**Results:**

The median direct medical expenses (DME) for the 117 patients were Chinese yuan (RMB) 65,182.66 (1 RMB = 0.14 USD). The median amount reimbursed by medical insurance was RMB 40,223.33, and the median out-of-pocket (OOP) payments after medical insurance reimbursement were RMB 21,938.82. The median subsidy amount was RMB 14,405.45, resulting in final OOP payments of RMB 8,167.13. Patients using bedaquiline, linezolid, and clofazimine received higher subsidies compared to non-users (*P* < 0.05). Logistic regression analysis showed that subsidy amounts below RMB 14,405.45 were significantly associated with adverse treatment outcomes (95%CI: 0.046–0.534, OR = 0.157, *P* = 0.003). The overall treatment success rate was 75.21%.

**Conclusion:**

MDR/RR-TB patients face a significant economic burden. The findings suggest that the subsidy policy may contribute to reducing out-of-pocket expenditures among MDR/RR-TB patients; however, further studies using stronger analytical designs are needed to evaluate its effectiveness. Optimizing subsidy criteria, raising subsidy levels and streamlining reimbursement procedures may help relieve patient financial stress, yet relevant benefits require verification via high-quality research.

**Supplementary Information:**

The online version contains supplementary material available at https://doi.org/10.1186/s12913-026-14998-x.

## Background

Drug-resistant tuberculosis (DR-TB) remains a major global health threat associated with high morbidity, mortality, prolonged treatment, and substantial costs [1, 2]. MDR/RR-TB treatment is expensive and challenging to access, and affected patients often face heavy economic burdens that exceed the coverage of current insurance and subsidy policies [3–7]. MDR/RR-TB imposes a heavy mental and economic burden on patients and their families and directly affects social stability and sustainable economic development [8]. In China, public health insurance provides these patients with 18–24 months of outpatient treatment, which offers very limited coverage [9]. These substantial barriers hinder patients with MDR/RR-TB from accessing and adhering to standard treatment protocols, necessitating robust supportive policies to assist patients in receiving and completing their treatment [10]. These policies include allocating special funds to equip designated MDR/RR-TB hospitals with antimicrobial susceptibility testing facilities and reagents, enhancing medical insurance benefits, and providing subsidies to patients [11, 12]. However, there is limited evidence on the impact of these policy implementations on the detection and treatment of MDR/RR-TB. Also, the equity issues inherent in the detection and treatment processes for patients with MDR/RR-TB have not been thoroughly studied. To promptly identify MDR/RR-TB cases in a timely manner, administer standardized and effective treatment, enhance patient treatment success rates, reduce further transmission of MDR/RR-TB, and contain the epidemic, Lishui City in Zhejiang Province launched the MDR/RR-TB prevention and treatment initiative across the city in September 2014. This initiative aligns with the implementation plan of the MDR/RR-TB standardized diagnosis and treatment project in Zhejiang Province (trial) and has established a management framework for MDR/RR-TB prevention and treatment [13]. However, the comprehensive effect of this policy on direct medical costs, out-of-pocket payments, patient satisfaction, and treatment outcomes has not been systematically evaluated. This study fills the evidence gap by using real-world data to assess the economic burden and policy effectiveness in a complete regional patient cohort. The findings will provide robust, locally applicable evidence to improve subsidy policies, reduce financial catastrophe, enhance treatment adherence, and support TB control goals.

Published studies cannot be directly generalized or applied to Lishui City, Zhejiang Province, because of substantial differences in local health insurance benefits, government subsidy standards, drug accessibility, healthcare delivery models, and socioeconomic conditions. Real-world, local evidence regarding the actual costs, subsidy effectiveness, patient satisfaction, and their impact on treatment outcomes remains scarce for this type of setting. Without context-specific data, it is difficult to optimize policies to truly alleviate patient burden and improve treatment success.

The study objectives are to describe the direct medical costs, medical insurance reimbursement, government subsidy amounts, subsidy ratios, and final OOP payments among patients with MDR/RR-TB in Lishui City, to evaluate patient awareness, satisfaction, and subjective evaluation of the adequacy of the current MDR/RR-TB subsidy policy, to explore the association between subsidy indicators (subsidy amount and subsidy ratio) and treatment outcomes, and to provide evidence-based suggestions for optimizing the MDR/RR-TB diagnosis and treatment subsidy policy.

From a health financing and TB control theoretical perspective, targeted government subsidies improve household financial protection by lowering patients’ out-of-pocket medical expenditure and mitigating the risk of catastrophic spending caused by expensive MDR/RR-TB treatment. Alleviated financial pressure reduces premature treatment discontinuation driven by unaffordable medical fees and improves continuous treatment adherence to full-course standardized anti-tuberculosis regimens. Improved adherence further promotes bacteriological conversion and contributes to superior final treatment outcomes.

## Methods

### Study design

This was a retrospective, observational, single-center study conducted in accordance with the STROBE (Strengthening the Reporting of Observational Studies in Epidemiology) guidelines. The study aimed to evaluate the direct medical costs, economic burden, and the impact of government subsidy policies among patients diagnosed with MDR/RR-TB.

### Study setting

The study was carried out at Lishui Hospital of Traditional Chinese Medicine, which serves as the designated hospital for the diagnosis, treatment, and management of MDR/RR-TB in Lishui City, Zhejiang Province, China. All eligible patients diagnosed between 2017 and 2022 were enrolled, and the retrospective survey was completed in January 2024.

### Study participants

This was a complete enumeration study that included all eligible patients; no sampling was performed. A total of 117 patients with MDR/RR-TB diagnosed and managed by the local MDR/RR-TB prevention and treatment expert panel from 2017 to 2022 were included in the study. The diagnosis was confirmed based on standard strain identification and drug susceptibility testing results.

#### Inclusion criteria

(1) Diagnosed with MDR/RR-TB; (2) Current permanent residence in Lishui City, Zhejiang Province; (3) Agreed to participate in the study and signed the informed consent form.

#### Exclusion criteria

(1) Patients who have never initiated anti-tuberculosis treatment; (2) Patients who have started anti-tuberculosis treatment but have not yet completed the treatment course.

### Research methods [14, 15]

Based on the data recorded by the hospital information system (HIS) regarding inpatient and outpatient treatment costs and medical insurance reimbursement, we assessed the direct medical expenses (DME) of the patients. This assessment encompassed the calculation of DME. DME is the costs associated with outpatient and inpatient care for patients with MDR/RR-TB, which included expenses related to laboratory tests, examinations, treatments, medications, and hospitalization. Notably, direct non-medical expenses such as those for nutrition and transportation were excluded from the analysis. The medical expenses incurred in Zhejiang Province were subtracted directly from the medical insurance system, with the government providing cash subsidies to patients. DME refers to the total amount of medical expenses before the deduction of insurance claims and government subsidies. OOP costs were defined as the actual direct medical expenses paid by the patient, specifically encompassing the total medical expenses excluding basic medical reimbursement and government subsidies [16]. All patients received provincial-level subsidies, with no financial support from the municipal or national governments.The costing perspective was the patient-level direct medical cost perspective.

The survey used a questionnaire developed by the research team (Supplementary File 1) to conduct a retrospective assessment of the participants. Relevant information was obtained by the investigators via face-to-face interviews. In cases where patients were deceased, family members provided responses on their behalf. Furthermore, relevant data on the diagnosis and treatment of patients with MDR/RR-TB, as well as information on subsidies and reimbursements, were obtained through consultation of the Tuberculosis Management Information System (TBIMS).

### Data collection

The study content encompassed an examination of the general status of the patients diagnosed with MDR/RR-TB, comprising of sociodemographic factors like age, sex, household registration, occupation, and personal monthly income. Also, it assessed the subsidies provided for the treatment costs of the patients and their perspectives on the subsidy policy. This included assessing whether patients comprehended the policy and determining their views on whether the subsidy ratio and scope were adequate. The subsidy ratio was calculated as the proportion of medical costs covered by subsidies, expressed as a percentage: subsidy ratio = (subsidy amount ÷ total medical expenses) × 100%. These assessments were conducted to assess the effectiveness of the current subsidy policy [14, 15]. 

The reimbursement ratio and scope were determined based on patients’ subjective assessments. Specifically: (1) The actual subsidy amount refers to the out-of-pocket cost after medical insurance reimbursement, but it was capped at 21,400 Chinese yuan (RMB), which may influence patients’ perception of whether the amount is sufficient. (2) The subsidy ratio we calculated was 19.22%, and patients are asked to evaluate whether such a percentage is sufficient. (2) The scope of the current DR-TB subsidy policy, including anti-TB drugs, hepatoprotective drugs, and TB-related examinations, was also subject to patient evaluation, particularly regarding whether additional costs should be covered. The effectiveness of the local MDR/RR-TB subsidy policy was evaluated across three domains: (1)Economic effect: reduction of patients’ out-of-pocket medical expenditure; (2)Subjective effect: patients’ awareness and satisfaction with subsidy implementation; (3)Clinical effect: whether higher subsidy amount/ratio is linked to improved treatment outcomes.

### Assessment criteria for treatment outcomes

Based on the WHO criteria adopted by TBIMS in China [17], the treatment outcomes of patients with MDR/RR-TB were recorded as follows:

#### Cure

Completion of the prescribed course of treatment, with no evidence of treatment failure, and negative results in at least three consecutive sputum mycobacterial cultures after the intensive treatment period (if no intensive treatment period is defined, it is recommended to be up to 8 months), with an interval of at least 30 days between each test.

#### Completion of the course of treatment

Completion of the prescribed course of treatment, with no evidence of treatment failure, but without obtaining the results of three consecutive sputum mycobacterial cultures with an interval of at least 30 days between each test after the intensive treatment period.

#### Treatment failure

Discontinuation of treatment or permanent change of at least two anti-TB drugs in the treatment regimen due to any of the following reasons.

- Sputum mycobacterial conversion was not achieved at the end of the intensive treatment period.
- Bacteriological relapse occurred in the continuation phase after sputum mycobacterial conversion.
- Evidence of acquired resistance to fluoroquinolones or second-line injectable drugs.
- Adverse drug reactions.

#### Death

Death due to any cause during the treatment process.

#### Loss to follow-up

Interruption of treatment for two consecutive months or more for any reason.

#### Successful treatment

Includes both cure and completion of the course of treatment.

#### Bacteriological conversion

Two consecutive sputum cultures were negative for mycobacteria, with at least 30 days between each test. The conversion time was defined as the date of the first negative sputum culture specimen collection.

#### Bacteriological relapse

After bacteriological conversion, the patient had two consecutive sputum cultures positive for mycobacteria, with an interval of at least 30 days between each test.

### Management of recall bias

The following measures have been implemented to minimize potential biases: (1) All survey tools used standardized, closed-ended questions. For example, monthly household income was categorized into predefined brackets (e.g., < RMB 2,000, RMB 2,000–4,000, > RMB 4,000) (1 RMB = 0.14 USD), and respondents were guided to recall key information based on critical time points (e.g., date of diagnosis, date of hospitalization). (2) Self-reported direct medical expenses (such as medication and hospitalization costs) were cross-checked with patients’ hospital billing records and health insurance reimbursement documents. A high level of consistency (89.7%) was observed. In cases of discrepancy, data were reconciled based on official medical records after discussion. (3) In 24 cases (20.51%), responses were provided by family members on behalf of the patient. For these proxy cases, additional verification steps were undertaken to ensure reliability: 100% of proxies were directly involved in patient care; 82% of cases retained relevant financial documents (e.g., invoices, loan agreements) related to medical expenses; and there were no statistically significant differences (*P* > 0.05) in key variables (e.g., total expenditure, number of hospitalizations) between proxy and non-proxy groups. These findings suggest that proxy response bias was limited and manageable.

### Statistical analysis

IBM SPSS Statistics 23.0 software was used for statistical analysis. Based on the Kolmogorov-Smirnov normality test, none of the continuous variables in this study conformed to a normal distribution; therefore, the median was used for description, and the Wilcoxon rank-sum test was used for comparisons between groups. Categorical variables were described by frequency and percentage, and the χ^2^ test was used for analysis. The impact of subsidy policies on treatment outcomes among patients with RR-TB was assessed using multivariate analysis based on a binary logistic regression model. A P-value of less than 0.05 (*P* < 0.05) was considered statistically significant.

## Results

### Basic patient information

A total of 122 MDR/RR-TB patients were screened from 2017 to 2022; 5 were excluded (4 declined treatment initiation, 1 received treatment outside Zhejiang Province), leaving 117 patients for final analysis.Among the 117 patients with MDR/RR-TB included in this survey, the age at diagnosis ranged from 15 to 88 years, with a median age of 55 years. The group comprised of 94 males and 23 females, yielding a male-to-female ratio of 4.09:1. Among the 117 patients, 114 had Lishui household registration and 3 had household registration outside Lishui City; however, all had permanent residence in Lishui City and met the inclusion criterion. Regarding occupational distribution, 77 patients were farmers, 18 had fixed incomes, and 22 were students or unemployed individuals without income. In terms of family economic status, 66 respondents (56.41%) had an average monthly income of less than RMB 2,000, 17 respondents (14.53%) had an income between RMB 2,000 and 4,000, and 34 respondents (29.06%) had an income > RMB 4,000. A significant majority, 99.15% (116/117) of the patients, had medical insurance, with only one patient from another province lacking insurance coverage. The treatment history indicated that 81 patients (69.23%) were treatment-naïve, while 36 patients (30.77%) were recurrent cases. Among the respondents, the hospitalization rate was 97.44% (114/117), and the comorbidity rate of diabetes mellitus was 11.97% (14/117).

### Expenses, subsidy and reimbursement during the treatment course of patients diagnosed with MDR/RR-TB

During the treatment course of the 117 patients with MDR/RR-TB, the DME amounted to RMB 65,182.66 (interquartile range [IQR]: RMB 41,061.61 to 84,227.97; Mean ± SD: RMB 72025.13 ± 4783.01). The hospitalization cost was RMB 17,186.16 (IQR: RMB 10,595.62 to 38,581.76; Mean ± SD: RMB 30375.34 ± 4159.57), and the outpatient cost was RMB 39,112.30 (IQR: RMB 14,681.14 to 58,928.43; Mean ± SD: RMB 41649.79 ± 2980.93). The medical insurance reimbursement covered RMB 40,223.33 (IQR: RMB 24,732.70 to 61,234.35; Mean ± SD: RMB 49207.77 ± 4272.54). After medical insurance reimbursement, the OOP payments were RMB 21,938.82 (IQR: RMB 14,001.68 to 29,928.78; Mean ± SD: RMB 22903.54 ± 1208.95). Post-subsidy median out-of-pocket (OOP) payments were RMB 8,167.13 (IQR: RMB 2,993.35 to 14,805.09; Mean ± SD: RMB 10541.57 ± 988.80).

### Implementation of subsidy policies

The awareness rate of the subsidy policy among the 117 respondents was 95.73% (112/117). Among these respondents, 81.20% (95/117) of patients with RR-TB received subsidies for their treatment costs. The main reasons for the 22 patients with RR-TB who did not receive subsidies were as follows: 10 of the 54 RR-TB patients diagnosed from 2017 to 2019 were not patients with MDR-TB, and only these patients were subsidized based on the policy at that time; also, 7 of the 12 patients with MDR/RR-TB who did not receive subsidies died within 1 month of diagnosis, and 5 refused anti-tuberculosis treatment or withdrew from treatment within 1 month despite multiple efforts to persuade them. Their treatment outcomes were recorded as “lost to follow-up.”

During the treatment period, the 117 patients with MDR/RR-TB received a total of RMB 1,446,349.95 in subsidies, with a median subsidy amount of RMB 14,405.45 (IQR: RMB 1,809.79 to 21,236.42) and a median subsidy ratio of 19.22% (IQR: 4.81% to 30.05%). The distribution of the subsidy amount and subsidy ratio among different groups is revealed in Table 1. The subsidy ratio for recurrent patients with MDR/RR-TB was lower than that for patients who were treatment-naïve (*P* = 0.017, Table 1). The amount of subsidy for patients with MDR/RR-TB who included BDQ, LZD, and clofazimine in their treatment regimen was higher than for those who did not use these medications (*P* < 0.05, Table 1). However, the subsidy ratio for patients with MDR/RR-TB who received LZD was higher compared to those who did not receive LZD (*P* < 0.05, Table 1). The subsidy amount and subsidy ratio for patients who were successfully treated were significantly higher than for those with adverse outcomes (*P* < 0.05, Table 1). Also, the subsidy amount and subsidy ratio for patients diagnosed with MDR/RR-TB between 2020 and 2022 were significantly higher than for those diagnosed between 2017 and 2019 (*P* < 0.05, Table 1).

**Table 1:** Distribution of the subsidy amount and ratio

**Types of patients**		**Cases**	**Subsidy amount/RMB**				**Subsidy ratio %**			
			Median	**Z**	**P**	Mean ± SD	Median	Z	P	Mean ± SD
Age	≥ 55 years	59	14047.98	-0.046	0.963	12893.93 ± 1141.39	18.97	-0.900	0.368	18.59 ± 1.86
	< 55 years	58	14468.49			13865.70 ± 1132.37	20.41			22.66 ± 1.96
Occupation	Farmer	77	15047.95	-1.701	0.089	14203.36 ± 986.06	19.30	-0.427	0.670	20.83 ± 1.65
	Non-farmer	40	9450.36			11688.11 ± 1358.08	17.15			19.94 ± 2.39
Monthly income/RMB	≥ 2000	51	13421.37	-0.529	0.597	12754.85 ± 1271.53	16.07	-0.789	0.430	18.69 ± 1.93
	< 2000	66	14819.44			13828.88 ± 1033.60	19.26			21.97 ± 1.88
Diabetes mellitus	Yes	14	13172.01	-0.160	0.873	11565.21 ± 2295.07	13.96	-0.236	0.813	18.55 ± 4.17
	No	103	14405.45			13604.93 ± 858.18	19.47			20.81 ± 1.44
Recurrence	Yes	36	10314.42	-1.677	0.094	13477.69 ± 1861.87	14.52	-2.394	0.017	18.40 ± 2.53
	No	81	14590.93			13316.41 ± 878.13	20.69			21.25 ± 1.60
Use of Bdq	Yes	17	20268.68	-2.002	0.045	15880.75 ± 1718.69	18.22	-0.799	0.424	21.00 ± 2.61
	No	100	12975.97			12880.42 ± 892.14	19.26			20.44 ± 1.54
Use of Lzd	Yes	42	17408.31	-2.269	0.023	14949.02 ± 1085.57	22.08	-2.229	0.026	22.56 ± 1.78
	No	75	12011.75			12328.49 ± 1107.55	14.68			19.22 ± 1.90
Use of Cs	Yes	107	14047.98	-0.606	0.544	13553.54 ± 843.11	19.22	-0.636	0.525	20.22 ± 1.33
	No	10	15160.45			11451.80 ± 2683.47	17.62			23.54 ± 6.79
Use of Cfz	Yes	31	19884.44	-2.125	0.034	15083.83 ± 1373.77	18.22	-0.626	0.531	18.59 ± 1.86
	No	86	12048.66			12652.79 ± 975.33	19.26			20.07 ± 2.07
Treatment outcomes	Successful treatment	88	17833.04	-5.045	< 0.001	14751.59 ± 841.62	22.84	-3.385	0.001	21.28 ± 1.43
	Adverse outcomes	29	943.10			5019.67 ± 1270.22	2.54			11.69 ± 2.89
Time of treatment initiation	2017–2019	54	8086.08	-3.664	< 0.001	9388.65 ± 1258.88	9.37	-3.418	0.001	14.41 ± 2.15
	2020–2022	63	18019.57			14910.52 ± 931.53	23.78			22.76 ± 1.53

Continuous variables were compared using Wilcoxon rank-sum test, while categorical variables were compared by χ^2^ test

RMB, Chinese yuan; Bdq, Bedaqualine; Lzd, linezolid; Cs, Cycloserine; Cfz, Chlorofazimine

Among the 117 respondents, 49.57% (58/117) of patients with MDR/RR-TB believed that the ratio and scope of subsidy and reimbursement were insufficient. Patients diagnosed with MDR/RR-TB between 2020 and 2022, as well as those who had successful treatment outcomes, were more satisfied with the ratio and scope of subsidy and reimbursement (*P* < 0.05, Table 2).

**Table 2:** The extent of reimbursement as perceived by patients with RR-TB

**Types of patients**		**Sufficient reimbursement ratio**		**Insufficient reimbursement ratio**		χ^2^	**P**	**Sufficient reimbursement scope**		**Insufficient reimbursement scope**		χ^2^	P
		Cases	Ratio %	Cases	Ratio %			Cases	Ratio %	Cases	Ratio %		
Sex	Male	45	47.87	49	52.13	1.249	0.264	47	50.00	47	50.00	0.035	0.852
	Female	14	60.87	9	39.13			12	52.17	11	47.83		
Age	≥ 55 years	28	47.46	31	52.54	0.420	0.517	29	49.15	30	50.85	0.077	0.781
	< 55 years	31	53.45	27	46.55			30	51.72	28	48.28		
Occupation	Farmer	40	51.95	37	48.05	0.208	0.648	43	55.84	34	44.16	2.644	0.104
	Non-farmer	19	47.50	21	52.50			16	40.00	24	60.00		
Monthly income/RMB	≥ 2000	25	49.02	26	50.98	0.072	0.789	24	47.06	27	52.94	0.410	0.522
	< 2000	34	51.51	32	48.49			35	53.03	31	46.97		
Comorbidity	Yes	5	35.71	9	64.29	1.377	0.241	7	50.00	7	50.00	0.001	0.973
	No	54	52.43	49	47.57			52	50.49	51	49.51		
Recurrence	Yes	15	41.67	21	58.33	1.597	0.206	16	44.44	20	55.56	0.745	0.388
	No	44	54.32	37	45.68			43	53.09	38	46.91		
Use of Bdq	Yes	8	47.06	9	52.94	0.090	0.764	11	64.71	6	35.29	1.622	0.203
	No	51	51.00	49	49.00			48	48.00	52	52.00		
Use of Lzd	Yes	25	59.52	17	40.48	2.169	0.141	25	59.52	17	40.48	2.169	0.141
	No	34	45.33	41	54.67			34	45.33	41	54.67		
Use of Cs	Yes	54	50.47	53	49.53	0.001	0.977	53	49.53	54	50.47	0.401	0.527
	No	5	50.00	5	50.00			6	60.00	4	40.00		
Use of Cfz	Yes	15	48.39	16	51.61	0.070	0.791	20	64.52	11	35.48	3.349	0.067
	No	44	51.16	42	48.84			39	45.35	47	54.65		
Successful treatment	Yes	51	57.95	37	42.05	8.047	0.005	54	61.36	34	38.64	16.986	< 0.001
	No	8	27.59	21	72.41			5	17.24	24	82.76		
Time of treatment initiation	2017–2019	19	35.19	35	64.81	9.320	0.002	21	38.89	33	61.11	5.341	0.021
	2020–2022	40	63.49	23	36.51			38	60.32	25	39.68		

RR-TB, rifampicin-resistant tuberculosis; RMB, Chinese yuan; Bdq, Bedaqualine; Lzd, linezolid; Cs, Cycloserine; Cfz, Chlorofazimine

### Effect of subsidy policies on the treatment outcomes of patients diagnosed with RR-TB

All 117 respondents completed the assessment of treatment outcomes. Among them, 88 patients had successful treatment outcomes (63 were cured and 25 had completed the course of treatment), while 29 patients had adverse outcomes (including 6 treatment failures, 10 deaths, and 13 lost to follow-up), resulting in a treatment success rate of 75.21%. Multivariate analysis using logistic regression revealed that a subsidy amount of less than RMB 14,405.45 was significantly associated with adverse treatment outcomes (*P* = 0.003, Table 3). However, a subsidy ratio of less than 19.22% was not significantly associated with adverse treatment outcomes (*P* = 0.588, Table 3).

**Table 3:** An examination using multiple variables to examine how subsidy policies impact the treatment outcomes of patients diagnosed with RR-TB

Subsidy policy	β value	Wald	S.E.	*P* value	OR value	95% CI of OR value
Subsidy amount < RMB 14405.45	-1.854	8.780	0.626	0.003	0.157	0.046–0.534
Subsidy ratio < 19.22%	-0.310	0.294	0.572	0.588	0.733	0.239–2.249

RR-TB, rifampicin-resistant tuberculosis; RMB, Chinese yuan; S.E., standard error; OR, odds ratio; CI, confidence interval

Adjusted covariates: age group, sex, occupation, monthly household income, new/retreatment status, and receipt of Bdq/Lzd/Cs/Cfz

## Discussion

The primary objective of this study was to evaluate direct medical costs, out-of-pocket expenses, government subsidy levels, and their associations with treatment outcomes among patients with MDR/RR-TB in Lishui City, Zhejiang Province, China. Our main findings revealed that low subsidy amount (< 14,405.45 RMB) was significantly associated with unfavorable treatment outcomes, whereas subsidy ratio showed no significant independent effect. Patients with MDR/RR-TB experienced heavy economic burden despite insurance coverage and local government subsidies. These real-world data highlight the critical role of subsidy policies in supporting successful treatment and underscore the need for region-specific policy optimization. WHO’s goal to end the global tuberculosis epidemic by 2035 has been impeded by the emergence of MDR/RR-TB [18]. Cost analysis studies conducted prior to 2015 indicate that the average cost of treating MDR/RR-TB ranges from USD 1,218 to USD 83,365 [19]. By 2016, the treatment costs for patients with MDR/RR-TB typically ranged between USD 2,000 and USD 20,000 [20]. During the treatment period of 117 MDR/RR-TB patients, the direct medical expenditure amounted to ¥65,182.66. These costs vary significantly between countries, and even within the same country, largely depending on the medication regimen implemented [21]. 

In this study, the medical insurance reimbursement ratio for patients with MDR/RR-TB, referring to the percentage of total treatment costs covered by public health insurance for patients with MDR/RR-TB in Lishui City, was 61.76%, the subsidy ratio was 19.22%, and the proportion of OOP payments was 12.81%, resulting in a total reimbursement ratio of 88.07%. This remains substantially below the government’s recommendation for free medical care. Approximately 49.57% of patients with MDR/RR-TB felt that the ratio and scope of subsidy and reimbursement were insufficient. With the multi-party payment model of medical insurance and financial subsidies in Zhejiang Province, the government supplements basic medical insurance reimbursements by covering part of the personal medical expenses of patients with MDR/RR-TB [22]. Despite this, the medical expenses remain high. The average DME for patients with MDR/RR-TB in this study were RMB 65,182.66 (USD 9,181), which is close to the average in Zhejiang Province (USD 10,491) [23], but lower than the average in Guangzhou in 2018 (USD 18,011.26) [24]. The average direct medical OOP payments in this study were approximately USD 1,150, which is lower than the average OOP payments in Zhejiang Province (USD 2,696) [23]. Given the similarity in DME, the reduction in OOP payments may be significantly influenced by the local medical insurance policy in Lishui City, Zhejiang Province, where the reimbursement ratio is higher than in other cities within the province.

Similar to nondrug-resistant TB, the economic burden of MDR/RR-TB arises from various factors, including delayed hospital visits, length of stay (LOS), poor patient adherence, interruption of treatment, and adverse drug reactions [25, 26]. Meanwhile, the high cost of MDR/RR-TB treatment is significantly related to the drugs used in anti-tuberculosis treatment regimens [27]. The findings of this study indicate that the subsidy amount for patients with RR-TB using BDQ, clofazimine, and LZD in their treatment regimen was significantly higher than for non-users. This may be attributed to the fact that few patients in this study used BDQ, LZD, and clofazimine [28]. Also, LZD was not covered by medical insurance, while the reimbursement ratio for BDQ and the second-line drug clofazimine was low.

In this study, the subsidy policy did not correlate with the age of the patient. The utilization rate of the emerging second-line drug BDQ for the treatment of MDR/RR-TB, referring to the proportion of patients who included BDQ in their treatment regimen out of the total cases, was found to be low, possibly due to the availability of the drug. The WHO has recently conditionally recommended a six-month regimen for patients with RR-TB, including BDQ, PA-824, LZD (600 mg), and moxifloxacin (BPaLM), instead of a nine-month or longer regimen [29]. Based on the new treatment protocol proposed by WHO, the utilization rate of BDQ is likely to increase, which may further elevate the treatment costs. A study in Korea found that new all-oral regimens including BDQ for the treatment of MDR-TB were estimated cost an additional 20,778 USD compared with conventional injectable-containing regimens [30]. However, BDQ is likely to be a cost-effective addition to existing treatment regimens [30, 31]. Shortening the treatment regimen allows for better patient adherence and more favorable treatment outcomes. Long-term treatment imposes both an economic and temporal burden on patients, whereas short-term continuous treatment not only reduces the economic burden but also decreases the number of patients receiving treatment at the same time, potentially reducing follow-up loss [32–35]. Patients receiving BDQ-containing regimens obtained significantly higher government subsidies compared with non-BDQ users, consistent with our statistical results. However, the elevated subsidy could not fully offset the substantial price premium of novel anti-TB agents including BDQ. The unit cost of BDQ is markedly higher than conventional second-line anti-tuberculosis medicines, resulting in a sharp rise in total direct medical expenditure for BDQ-treated patients. Although local subsidy policy prioritizes reimbursement for expensive new drugs and raises the upper limit of financial support for BDQ recipients, the incremental subsidy increment is lower than the extra drug expenditure brought by BDQ. Consequently, after deducting medical insurance reimbursement and government subsidy, BDQ users still bear higher residual out-of-pocket payments than patients treated with traditional regimens, which means the out-of-pocket gap is further widened for this subgroup.

From a policy perspective, the existing subsidy effectively mitigates part of the extra economic burden induced by expensive novel anti-TB drugs and avoids catastrophic expenditure for most BDQ patients, yet current subsidy standards fail to fully cover the price premium of new medications. To further reduce patients’ personal spending, targeted subsidy tilt or centralized bulk procurement for BDQ is required to narrow the persistent OOP gap among BDQ receivers.

The treatment success rate among the 117 patients was 75.21%. This study confirmed that patients diagnosed with RR-TB having successful treatment outcomes expressed greater satisfaction with the ratio and scope of the subsidy and reimbursement, and that both the amount and ratio of the subsidy were significantly higher than those with adverse outcomes. For patients who never received anti-tuberculosis treatment, the subsidy amount was zero. Also, the subsidy amount was relatively lower for patients who interrupted treatment due to anti-tuberculosis drug-induced adverse reactions, died, or were lost to follow-up during the treatment course, as they did not complete the prescribed anti-tuberculosis treatment.

With the end of the Global Fund for Tuberculosis program in China in June 2014, the economic burden on patients with DR-TB in areas lacking government subsidies has increased significantly. From previous research, we have discovered that following the withdrawal of global funds from China, domestic capital investment has decreased, and the supply of second-line drugs cannot be effectively ensured [36]. In recent years, the basic medical insurance network of China, encompassing basic medical insurance for urban employees, the new rural cooperative medical insurance, and medical insurance for urban residents, has made substantial progress, covering more than 1.3 billion people (approximately 95% of the total population) as of 2018 [37]. Concurrently, the government provides financial subsidies for patients diagnosed with MDR/RR-TB through designated tuberculosis hospitals or local centers for disease control and prevention [23]. 

The investigation revealed that 56.41% (66/117) of patients had a personal monthly average income of less than 2,000 RMB. Patients diagnosed with MDR/RR-TB generally faced poor family economic conditions. The extended duration and high cost of treatment imposed a significant economic burden, leading some patients to delay or abandon treatment, which adversely impacted not only the patients themselves but also their families and society at large. Reducing the economic burden on patients has been known to effectively increase treatment adherence [4, 38]. Also, within the context of hierarchical medical care policy, the medical insurance reimbursement ratio in Lishui City, Zhejiang Province has decreased with the level of hospital enhancement, and restrictions on insurance reimbursement remain. This is a significant departure from the previous practice of providing anti-tuberculosis drugs free of charge and can negatively affect treatment compliance. Therefore, it is recommended that the government further strengthen diagnosis and treatment subsidies, expand the eligible population, increase the subsidy amount, and broaden the scope of subsidies. A more flexible and effective diagnosis and treatment subsidy program tailored to different treatment regimens should be formulated. Also, the reimbursement and subsidy process for patients with MDR/RR-TB should be optimized to enhance efficiency [39]. 

For MDR/RR-TB patients, the standard treatment duration is 18–24 months, with follow-up visits required at least once per month. Patients who achieve successful treatment outcomes generally have longer follow-up periods compared to those with unfavorable outcomes such as loss to follow-up, treatment failure, or death. Consequently, their direct medical expenses (DME) tend to be higher. After medical insurance reimbursement, the out-of-pocket costs increase accordingly, leading to higher subsidy amounts. Therefore, patients with successful treatment outcomes receive more financial subsidies. In addition, subsidies are issued quarterly and require patients to provide relevant DME invoices. Some patients with poor outcomes, such as those lost to follow-up, experiencing treatment failure, or who have died, may be unable to provide the necessary invoices, resulting in their failure to receive the corresponding subsidies.

This study has several limitations. First, the single-center enrollment restricts the generalizability of findings to other regions with distinct medical resource and subsidy policies. Second, only direct medical expenses were analyzed; indirect costs such as travel, accommodation and lost household income were not included, which underestimates total household economic burden. Third, monetary costs spanning 2017–2022 were not adjusted for inflation, potentially affecting longitudinal cost comparison across study years. Fourth, we did not perform standardized calculation of catastrophic healthcare expenditure based on WHO criteria and cannot quantify the prevalence of catastrophic financial burden among enrolled patients. Fifth, preset inclusion and exclusion criteria excluding untreated or incomplete-treatment patients may result in selection bias. Sixth, despite data cross-verification with hospital administrative systems and standardized questionnaire design, partial proxy-reported information from family members still carries residual recall bias. Seventh, critical reverse causality cannot be ruled out in this observational study: impoverished patients tend to receive higher subsidies due to poor financial status, whereas low income itself increases the risk of unfavorable treatment outcomes. Accordingly, the significant association between higher subsidy amount and improved treatment success only reflects statistical correlation rather than causal effect of the subsidy policy. We also failed to collect detailed data on heterogeneous impacts of various comorbidities and extra high-cost medical insurance benefits. In addition, the relatively small sample size limits statistical robustness. Future large-scale multicenter prospective research is required to verify our conclusions and comprehensively evaluate the real effectiveness of local subsidy policy.

## Conclusion

Our findings underscore the significant economic burden faced by patients diagnosed with MDR/RR-TB, which is notably influenced by the costs associated with anti-tuberculosis therapy. The findings suggest that the subsidy policy may contribute to reducing out-of-pocket expenditures among MDR/RR-TB patients; however, further studies using stronger analytical designs are needed to evaluate its effectiveness. A subset of patients still cannot access subsidy support, and heavy financial pressure or adverse drug reactions may trigger delayed, interrupted or abandoned treatment, leading to poor treatment outcomes.To address these challenges, we propose expanding subsidy coverage, improving access to new second-line anti-TB medicines, appropriately raising subsidy amounts and simplifying reimbursement workflows. These proposed adjustments may help ease patients’ financial burden, yet their actual effects need to be confirmed by subsequent rigorous research.

## Supplementary Information

Below is the link to the electronic supplementary material.

Supplementary Material 1

## Data Availability

All data generated or analysed during this study are included in this article. Further enquiries can be directed to the corresponding author.

## Abbreviations

MDR/RR-TB: Multi-drug resistant and rifampicin-resistant tuberculosis
DME: Direct medical expenses
OOP: Out-of-pocket
DR-TB: Drug-resistant Tuberculosis
RR-TB: Rifampicin-resistant tuberculosis
MDR-TB: Multidrug-resistant tuberculosis
WHO: World Health Organisation
HIS: Hospital information system
TBIMS: Tuberculosis Management Information System
TB: Tuberculosis
IQR: Interquartile range
BPaLM: Bedaquiline, pretomanid, linezolid and moxifloxacin
Bdq: Bedaqualine
Lfx: Levofloxacin
Mfx: Moxifloxacin
Cfz: Chlorofazimine
LOS: Length of stay
DST: Drug susceptibility tests
Lzd: linezolid
