# Supplementary material for: Economic burden of MDR/RR-TB and implementation of a treatment subsidy policy in Lishui City, China: a retrospective study following strobe guidelines
Source: BMC Health Serv Res. 2026 Jun 23;26:1249. doi: 10.1186/s12913-026-14998-x (PMC13556018; doi:10.1186/s12913-026-14998-x)
Supplement: Supplementary file 1 [file 12913_2026_14998_MOESM1_ESM.docx]

**The Survey Questions are as follows:**

1. Do you think the current DR-TB subsidy policy's coverage scope (including anti-TB drugs, hepatoprotective drugs, and TB-related examination items) is sufficient?

[Yes; No (Please specify what additional items you think should be covered________)]

2. Do you think the current subsidy amount under the DR-TB policy (out-of-pocket costs after insurance reimbursement, capped at 21,400 RMB) is sufficient?
[Yes; No (Please specify the amount you believe should be covered: ________ RMB)]

3. Do you think the current subsidy ratio under the DR-TB policy is sufficient?
[Yes; No (Please specify the percentage you believe should be covered: ________ %)]

The patient's satisfaction is primarily based on their subjective assessment of the subsidy ratio and coverage scope. The following question was asked:

Are you satisfied with the current subsidy policy for drug-resistant tuberculosis (DR-TB)?

(Yes; No).

**Questionnaire on medical subsidy policy for MDR/RR-TB patients in Lishui, Zhejiang province**

1. Gender: □Male	□Female
2. Date of birth: YYYY-MM-DD
3. Domicile address:

□Lishui city, Zhejiang province	District (county, city)

□	province	city	District (county, city)

4. Occupation:

□Farmer

□Company employees

□Workers

□Retirees

□Migrant workers

□Individual

□Students

□Unemployed

□Other

5. Do you know you have drug-resistant tuberculosis?

□Yes

□No

6. Have you been hospitalized for drug-resistant tuberculosis？

□Yes

□No

7. Do you have diabetes？

□Yes

□No

8. Do you have any other diseases (besides diabetes)？

□Yes （Please fill in the name of the disease	)

□No

9. 9. Do you have medical insurance?

□Yes

□No

10. What is your monthly income:

□<2000 yuan/month

□2000-4000 yuan/month

□>4000 yuan/month

11. Do you or your family members know that the Zhejiang provincial government has a subsidy policy for drug-resistant tuberculosis patients?

□Yes

□No (If No was selected, investigators were asked to inform the respondents of the current subsidy policy for drug-resistant tuberculosis in Zhejiang province in detail, and to conduct a 12-16 question survey)

12. Are you satisfied with the current subsidy policy for drug-resistant TB?

□Yes

□No

13. Do you think the exemption scope of the current subsidy policy for drug-resistant tuberculosis (including anti-tuberculosis drugs, liver protective drugs, and tuberculosi-related tests) is sufficient?

□Yes

□No (Please state what else you think you need to reduce	)

14. 14. Do you think that the amount of reduction and exemption of the current subsidy policy for drug-resistant tuberculosis (the out-of-pocket part after medical insurance reimbursement, but the upper limit is 21400 yuan) is sufficient?

□Yes

□No（Please tell me how much you think you need to reduce	)

15. Do you think the current subsidy policy reduction ratiofor drug-resistant tuberculosis is sufficient?

□Yes

□No(Please indicate your opinion on the need for a reduction of _%)

16. Do you have any further explanation or clarification regarding the above issue?

□No

□Yes (Please fill in the description	)

Investigator:

Date: YYYY-MM-DD
